# Cost‐effectiveness of sunflower oil fortification with vitamin A in Tanzania by scale

**DOI:** 10.1111/mcn.12720

**Published:** 2019-05-31

**Authors:** Dylan Walters, Edna Ndau, Nadira Saleh, Theobald Mosha, Susan Horton

**Affiliations:** ^1^ Canadian Centre for Health Economics, Institute of Health Policy, Management and Evaluation University of Toronto Toronto Ontario Canada; ^2^ Nutrition International Ottawa Ontario Canada; ^3^ Department of Nutrition and Consumer Sciences Sokoine University of Agriculture Morogoro Tanzania; ^4^ MEDA Waterloo Ontario Canada; ^5^ Department of Food Technology, Nutrition and Consumer Sciences Sokoine University of Agriculture Morogoro Tanzania; ^6^ School of Public Health and Health Systems University of Waterloo Waterloo Ontario Canada

**Keywords:** child nutrition, cost‐effectiveness, fortification, sunflower oil, vitamin A

## Abstract

In 2011, Tanzania mandated the fortification of edible oil with vitamin A to help address its vitamin A deficiency (VAD) public health problem. By 2015, only 16% of edible oil met the standards for adequate fortification. There is no evidence on the cost‐effectiveness of the fortification of edible oil by small‐ and medium‐scale (SMS) producers in preventing VAD. The MASAVA project initiated the production of sunflower oil fortified with vitamin A by SMS producers in the Manyara and Shinyanga regions of Tanzania. A quasi‐experimental nonequivalent control‐group research trial and an economic evaluation were conducted. The household survey included mother and child pairs from a sample of 568 households before the intervention and 18 months later. From the social perspective, the incremental cost of fortification of sunflower oil could be as low as $0.13, $0.06, and $0.02 per litre for small‐, medium‐, and large‐scale producers, respectively, compared with unfortified sunflower oil. The SMS intervention increased access to fortified oil for some vulnerable groups but did not have a significant effect on the prevention of VAD due to insufficient coverage. Fortification of vegetable oil by large‐scale producers was associated with a significant reduction of VAD in children from Shinyanga. The estimated cost per disability‐adjusted life year averted for fortified sunflower oil was $281 for large‐scale and could be as low as $626 for medium‐scale and $1,507 for small‐scale producers under ideal conditions. According to the World Health Organization thresholds, this intervention is very cost‐effective for large‐ and medium‐scale producers and cost‐effective for small‐scale producers.

**Key messages**

- Vitamin A fortification of sunflower oil by small‐ and medium‐scale producers may have increased access to fortified oil for some vulnerable groups in Tanzania.
- The mandatory fortification of oil with vitamin A was associated with significant reductions in the prevalence of vitamin A deficiency (VAD) in children and women in a region that commonly consumes vegetable oil fortified by large‐scale producers.
- The cost per disability‐adjusted life years averted of vitamin A fortification of sunflower oil was $281 for large‐scale producers and could be as low as $626 and $1,507 for medium‐ and small‐scale producers.
- Evidence suggests that vitamin A fortification of sunflower oil by large‐ and medium‐scale producers is very cost‐effective in preventing VAD.

## 1 INTRODUCTION

Globally, vitamin A deficiency (VAD) was associated with 105,700 child deaths in 2013 and affects another 90 million children and 19 million women (Black et al., 2013; Stevens et al., 2015). The highest levels of deficiency are in the Central and Eastern Africa regions, where nearly half of all children are affected (Stevens et al., 2015). The development of a sustainable agricultural system is the desired approach in the long run to improve food security, diversity of diets, and reduce the burden of VAD across low‐ and middle‐income countries (LMICs; Stevens et al., 2015). In recent decades, governments have invested in the scale‐up of vitamin A supplementation (VAS) to nearly universal coverage in LMICs (United Nations Children's Fund, 2016). The ongoing debate over the sustainability of VAS programmes is cause for more research as to the potential of mass food fortification with vitamin A to prevent VAD (Klemm, Palmer, Greig, Engle‐Stone, & Dalmiya, 2016; Wirth et al., 2017).

Using edible cooking oil as a food vehicle for vitamin A fortification is very appropriate because the fat‐soluble form of the vitamin is inexpensive; the oil protects the vitamins from oxidation and is nearly universally consumed by households in some regions with a high burden of VAD (World Health Organization & Food and Agriculture Organization, 2006). The World Health Organization (WHO) guidelines state that vitamin A fortification using margarine and oil food vehicles is efficacious as well as historically effective in developed countries, but that more effectiveness research is needed before issuing a recommendation for scale‐up in LMICs (Das, Salam, Kumar, & Bhutta, 2013; World Health Organization, 2017). Twenty‐eight LMICs have enacted mandatory fortification laws and standards for edible oil since the turn of the millennium (Food Fortification Initiative, Global Alliance for Improved Nutrition, Iodine Global Network, & Micronutrient Forum, 2017).

In Tanzania, VAD affects 33% or 3.3 million children and 37% or 4.9 million women (National Bureau of Statistics Tanzania & International Classification of Functioning, Disability, and Health [ICF] Macro, 2011). In 2011, the government of Tanzania passed a mandatory legislation that requires all edible oil produced by large‐scale enterprises in the country to be fortified with vitamin A (Tanzania Food and Drugs Authority, 2011). Vitamin A fortification was projected to reduce the prevalence of VAD by 30% (National Food Fortification Alliance, 2009). The required fortification level in the standards was 16–28 mg/kg of retinyl plamitate, which began to be enforced in late 2014 (Global Alliance for Investment in Nutrition, 2017; Randall, 2016; Tanzania Bureau of Standards, 2010). The East African Community, which includes Tanzania, also enacted a standard in 2014 specifying a higher fortification level of 20–40 mg/kg retinyl palmitate and a recommended factory level of 35 mg/kg (East African Community, 2017). Small‐scale producers were given a grace period of 2 years, until 2016, to comply. Fortification programmes in sub‐Saharan Africa tend mainly to reach consumers who buy food processed by large‐scale producers. Even though 54% of edible oil consumed in Tanzania in 2015 was fortified by large‐scale producers (Aaron et al., 2017), only 16% of the edible oil met the standards for being adequately fortified (Global Alliance for Investment in Nutrition & Tanzania National Bureau of Statistics, 2015).

Amid this period of change in national fortification policy, the MASAVA project was established to initiate and build capacity of small‐ and medium‐scale (SMS) producers in the production of unrefined sunflower oil fortified with vitamin A. The project was implemented by Mennonite Economic Development Associates (MEDA), the University of Waterloo, and the Sokoine University of Agriculture. The project partnered with three SMS producers in processing and distributing vitamin A fortified sunflower oil to over 200 hundred retailers in the Manyara and Shinyanga regions. This project included a research component designed to evaluate the effectiveness of fortification in preventing VAD in children under 5 years of age and lactating mothers. Cumulative production of the fortified oil exceeded 140,000 L over the 18‐month period of project support between 2015 and 2017. Other related publications contain additional information about the MASAVA project (Horton, Saleh, & Mosha, 2017; Walters et al., 2018).

The Manyara and Shinyanga regions, each containing approximately 1.5 million inhabitants, have poverty levels that are higher than the national average (Ministry of Health, Community Development, Gender, Elderly, and Children [MoHCDGEC] et al., 2016). Only 51% of children under 5 years of age consumed vitamin A rich foods in 24 hr preceding the survey, and only 27% of children (6–59 months) received a vitamin A supplement in the 6 months before the survey in Manyara (MoHCDGEC et al., 2016). In Shinyanga, there was higher consumption of vitamin A rich foods by children (85%) and higher coverage of vitamin A supplements (37%) in 6 months before the survey (MoHCDGEC et al., 2016). This study occurred within the context of a natural experiment where households in one region (Manyara) preferred sunflower oil, commonly produced predominantly in this region by SMS enterprises, whereas the other region (Shinyanga) preferred vegetable oil produced by large‐scale fortification programmes, which by happenstance, was also being scaled‐up over the same period. In 2010, 82% of households in Manyara used sunflower oil as their primary cooking oil whereas 64% of households in Shinyanga used vegetable oil (predominantly imported palm olein; National Bureau of Statistics Tanzania & ICF Macro, 2011). With the lack of dietary diversity, low coverage of vitamin A supplementation, and high levels of poverty, households in these two regions may be considered highly vulnerable to being deficient in vitamin A. At the time of the 2010 Demographic and Health Survey (DHS), the prevalence of VAD was 44 and 37% in children under 5 years of age in Manyara and Shinyanga, respectively (National Bureau of Statistics Tanzania & ICF Macro, 2011).

The prospective economic evaluation on fortification presented to the government of Tanzania (National Food Fortification Alliance, 2009) estimated that VAD alone was responsible for $32 million in economic losses each year, while investing in the scale‐up of vitamin A fortification could generate an economic return of up to $36 for every dollar invested. However, the evaluation relied on hypothetical projections of effectiveness and costing data from large‐scale producers only, which does not account for the potential role of SMS producers to produce fortified oil.

This study seeks to address several policy questions related to vitamin A fortification in Tanzania: (1) What are the determinants of access to fortified oil by vulnerable households?; (2) What is the incremental cost of the production and distribution of fortified oil by SMS producers compared with large‐scale producers?; and (3) Is the fortification of oil with vitamin A, by either small‐, medium‐, or large‐scale producers, cost‐effective in the reduction of VAD in children and women?

## 2 METHODS

### 2.1 Research trial design

The research component of the MASAVA project included a quasiexperimental nonequivalent control‐group trial, with one control and three intervention districts in each of the Manyara and Shinyanga regions. Control districts were selected based on the municipal divisions. Children 6–59 months of age and their lactating mothers were the units of observation for this research trial. Local nutrition officers and health care personnel at health centres helped identify eligible mothers to participate in the household survey. Eligible mothers were randomized within districts from stratified groups by age, geographic area, and income to obtain a heterogeneous sample. Inclusion criteria for the index child in each household were being between 6 and 59 months of age and being the oldest child under five of a lactating mother (at baseline).

The household survey questionnaire contained multiple modules. The DHS programme methodology served as a template for the modules on household demographics, wealth index (including property, housing facilities, and consumer goods), and mother and child nutrition and feeding behaviours (Measure Demographic and Health Survey, 2014). The survey also included modules on food security, food consumption, and dietary diversity (Food and Agriculture Organization, 2013) as well as oil fortification coverage based on the Global Alliance for Improved Nutrition's fortification assessment coverage tool (Aaron et al., 2017). A Helen Keller International questionnaire was used as the template for the vitamin A knowledge, attitude, and practices module (Helen Keller International, 2012). Data collection also included dried blood finger prick samples for the mother and child pair, anthropometric measurements of the index child (height and weight), and a sample of the available cooking oil from each household. All modules were collected both at baseline and at end line, with the exception of the asset variables. The National Institute for Medical Research in Tanzania and the University of Waterloo both provided Research Ethics Board approval. The baseline data for this study were collected between May and July 2015, and the end line survey conducted approximately 18 months later between November 2016 and January 2017. Other related publications and reports contain additional information about the MASAVA research trial (Horton et al., 2017; Walters et al., 2018).

### 2.2 Statistical methods

The household survey sample size calculation, which estimated that a sample size of 385 would be required, assumed a maximum variability in the population (*p* = 0.50), a 5% margin of error, and a 95% confidence interval (CI; Wu, Corbett, Horton, Saleh, & Mosha, 2019). To account for the high uncertainty of attrition at end line, the sample size was inflated to include 568 households. Assuming an expected mean retinol level of 14.84 μg/ml in children of the group unexposed to fortification (based on the baseline mean retinol in Manyara), the unbalanced sample size design (*N* ratio = 0.55) provided sufficient power to detect a 10% change in mean serum retinol when comparing the intervention and control groups (*β* = 0.88). Among the 568 households that participated in the baseline survey, there were 366 mother and child pairs in the intervention group and 202 in the control group. Attrition in the total number of households participating in the survey at end line compared with baseline was 13%. Moreover, dried blood samples could not be collected at end line for 21% of mothers and 24% of children surveyed at baseline; therefore, a total of 412 children participated at end line. Comparison of the participant characteristics and vitamin A status of attrited versus nonattrited children suggested that it was unlikely that attrition introduced any systematic bias (Walters et al., 2018).

A multivariate regression analysis of access to fortified oil was undertaken using the end line data. Because consumption of adequately fortified oil was small at baseline, we do not present these results here. The underlying theoretical model was informed by the prevailing conceptual frameworks for the determinants of malnutrition (Bhutta et al., 2013; Black et al., 2013) and economic models of health investment and household allocation of resources in low‐resource settings (Alderman, Chiappori, Haddad, Hoddinott, & Kanbur, 1995; Strauss & Thomas, 1998). Both ordinary least squares (OLSs) and quantile regression analyses were used to examine the effect of independent variables representing geography, socio‐economic status, food and oil consumption, and knowledge of vitamin A, on the dependent variable representing household access to fortified oil, namely, oil retinol levels in milligram per kilogram measured from the samples collected. Independent variables in the model included household location (whether the household was located in an intervention or control district), region, and urban or rural, as well as a household wealth index score, maternal dietary diversity, knowledge of the benefits of vitamin A, and primary oil‐type consumed. The OLS regression only measures the effect of the independent variables on the dependent variable at the mean of the distribution, whereas the quantile regression analysis allows for a more flexible, non‐linear relationship between independent and dependent variables at the median and various percentiles of interest (Hill, Griffiths, & Lim, 2011). In the quantile regression, the dependent variable quantile rank is the proportion of values in the oil retinol level distribution that are greater or equal to the percentile. The quantile regression was conducted for the 10th, 25th, 50th, 75th, and 90th percentiles, and statistical significance at *p* < 0.05 and 0.01 levels was reported. Stata V.14.2 was used for all univariate, bivariate, and multivariate analyses.

### 2.3 Costing and cost‐effectiveness analysis

The Fiedler and Afidra (2010) economic evaluation of vitamin A fortification in Uganda and the relevant guidelines for the economic evaluation in health programmes informed the methodological design of this costing and cost‐effectiveness analysis (Bill and Melinda Gates Foundation, 2014; Drummond, Sculpher, Torrance, O'Brien, & Stoddart, 2005). The costing analysis of fortified oil in Tanzania compared with unfortified oil, and within each of the three producer scale categories, was conducted from the societal perspective for a 1‐year time period and included both private and public sector costs. Private sector cost components included start‐up costs, which were annuitized, for the purchase and installation of equipment to mix the fortificant with the oil, initial training of staff, and redesign of oil labels. Private sector recurrent costs for the purchase of the vitamin A premix, additional energy use and labour for mixing, quality assurance testing, and packaging of fortified oil if different from unfortified oil were included. Management and distribution fees as well as the government value‐added tax were included as a proportion of the direct costs where applicable. Monthly monitoring data provided by the MASAVA project SMS partners and consultant reports were the primary sources of fortification unit‐cost data (Market Axis Limited, 2016; Randall, 2016). Public sector costs for regulation, social marketing, monitoring, and programme management were crudely estimated based on the previous estimates from a prospective economic evaluation of fortification in Tanzania (National Food Fortification Alliance, 2009). All costs were expressed in 2017 U.S. dollars (US$), and the 90‐day average exchange rate on December 5, 2017, of 2,325 Tanzania shillings (TZS) to one US$ was used for necessary conversions. 1Currency exchange rates retrieved from http://www.xe.com.


For the cost‐effectiveness analysis, the estimated health effect was drawn from the results of a differences‐in‐differences (DIDs) regression analysis of the reduction of the prevalence of VAD in children and women (Horton et al., 2017; Walters et al., 2018; Walters, 2018). This study assumed a 1:1 serum retinol to retinol‐binding protein ratio (e.g., the threshold for VAD was below 0.7 or 14.44 μg/ml in children and below 1.05 or 26.04 μg/ml in women; Namaste et al., 2017). This health effect was factored into a population‐attributable fraction equation using the published relative risks of mortality from childhood diarrhoea and measles associated with VAD (Stevens et al., 2015) and global burden of disease data on morbidity associated with VAD to calculate the hypothetical total number of child deaths and disability‐adjusted life years (DALYs) averted (Institute for Health Metrics and Evaluation, 2017). The total number of DALYs averted due to fortification of oil with vitamin A was then combined with the above‐mentioned estimates on the incremental cost of fortification to estimate the cost per DALY averted for each of small‐, medium‐, and large‐scale producers. The health effect of fortification observed in the Shinyanga region, which relied more on consumption of oil fortified by large‐scale producers, was extrapolated to the entire country for this calculation.

Sensitivity analysis was also conducted on costing and cost‐effectiveness results by varying values for key cost drivers including production volume, the cost of fortification equipment, and the cost of not using recycled oil containers. The health effect estimate of fortification was varied to both low and higher values derived from the DID regression analysis (Walters et al., 2018).

## 3 RESULTS

### 3.1 Determinants of access to fortified oil

**Figure 1:**
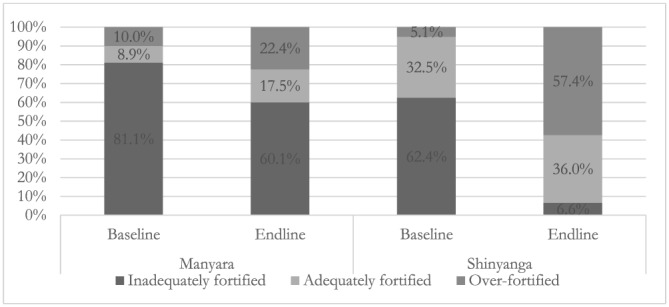
Percent of households whose oil sample met the Tanzania Bureau of Standards fortification criteria for “adequately fortified” (36.6–73.3 mg/kg retinol, which is equivalent to 20–40 mg/kg of retinyl palmitate

**Figure 2:**
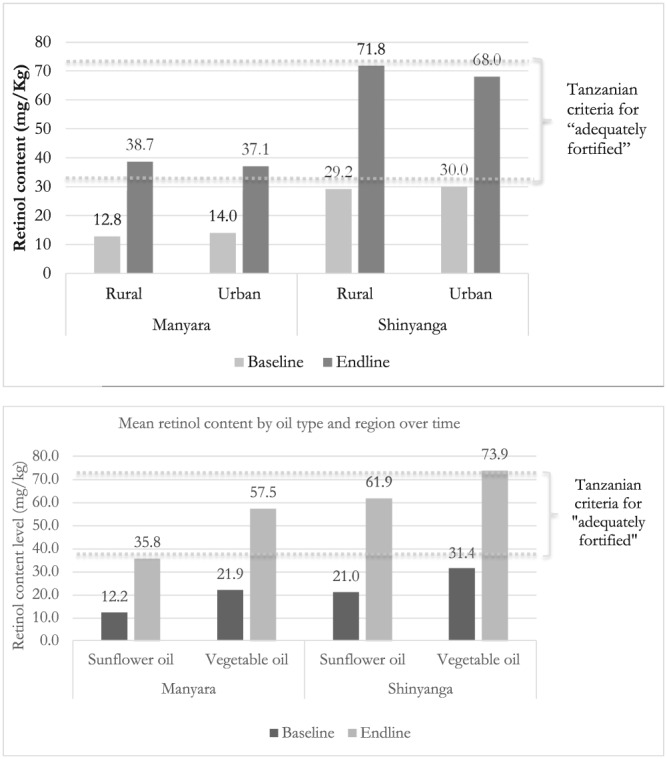
(a) Mean retinol level in edible oil of MASAVA sample by household location. (b) Mean retinol level in edible oil of MASAVA sample households by type of oil

At baseline in 2015, only 17% of the MASAVA cooking oil samples (Figure 1) were adequately fortified (e.g., between 36.6 and 73.3 mg/kg of retinol, which is equivalent to 20–40 mg/kg of retinyl palmitate), which is consistent with the national survey estimate of 16.3% in the same year (Global Alliance for Investment in Nutrition & Tanzania National Bureau of Statistics, 2015). There are stark differences in the type of oil consumed by households in the Manyara and Shinyanga regions as follows: At the end of the study period, 96% of households in Manyara consumed sunflower oil whereas 63% of households in Shinyanga consumed vegetable oil (including palm oil), and only 33% consumed sunflower oil. In Manyara, the share of households with adequately fortified oil increased rapidly from 9% at baseline to 18% at end line, and that with overfortified oil (i.e., >73.3 mg/kg of retinol) increased from 10 to 22% over the study period. In Shinyanga, the share of households with adequately fortified oil increased from 32 to 36%, and that with overfortified oil increased from 5 to 57%. In the whole sample, the mean oil retinol level was 49.09 mg/kg (*SD =* 27.75). The mean retinol level in household cooking oil at end line was 38 mg/kg in Manyara and 71 mg/kg in Shinyanga (Figure 2a,b), both within the national standards criteria range for adequate fortification (East African Community, 2017). The large increase in overfortification of oil and higher mean oil retinol level in Shinyanga were likely due to overcompensation by large‐scale producers attempting to adhere to the newly enforced fortification standards.

The OLS regression analysis on the determinants of access to fortified oil, represented by retinol content in household oil, found large positive associations between oil retinol levels and living in the intervention districts (13.22; 95% CI: 9.12, 17.31, *p* < 0.01), living in the Shinyanga region (22.24; 95% CI: 16.48, 27.99, *p* < 0.01), and consuming vegetable oil instead of sunflower oil (13.34; 95% CI: 7.26, 19.42, *p* < 0.01). Living in urban areas was negatively associated with retinol levels (−5.35, 95% CI: −9.78, −0.93, *p* < 0.05).

**Figure 3:**
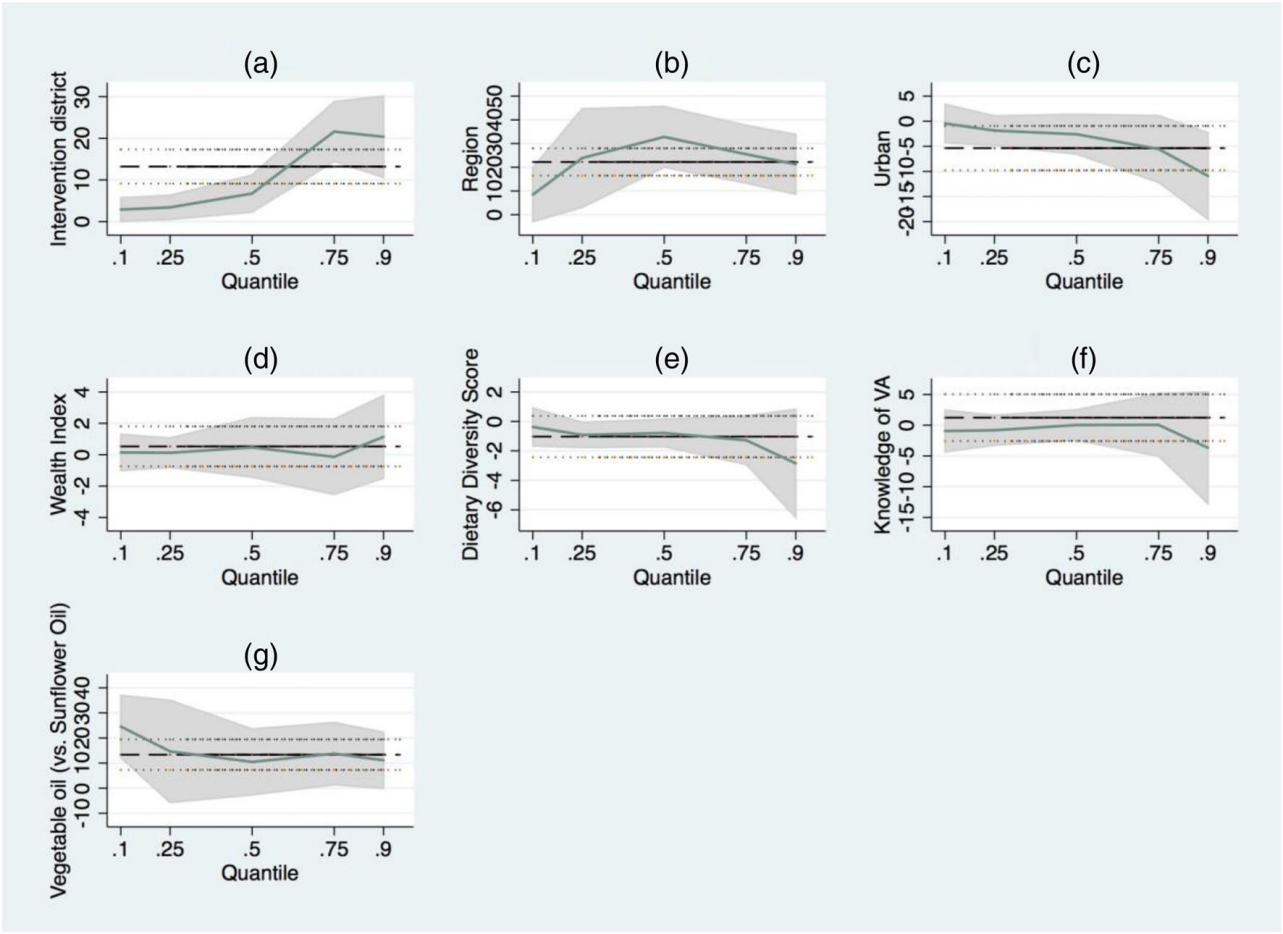
Graphical illustration of the quantile regression results: Coefficients (curved line) and 95% confidence interval (grey zone) as well as ordinary least square (OLS) coefficient (horizontal line) and OLS confidence interval (dashed horizontal lines)

In the quantile regression analysis, living in an intervention district was positively associated with higher retinol levels at each of the five percentiles examined. Living in the Shinyanga region had positive associations with higher retinol levels at all except the 10th percentile (8.48; 95% CI: −4.45, 21.42, *p* = 0.82), and the magnitude of the effect of living in the Shinyanga region was largest at the 25th, 50th, and 75th percentile as shown in Figure 3. There were also positive associations between vegetable oil consumption and higher retinol levels at all five quintiles, but the effects were largest and significant only at the 10th (24.56; 95% CI: 12.69, 36.44, *p* < 0.01), 75th (13.77; 95% CI: 3.27, 24.27, *p* < 0.5), and 90th percentiles (11.12; 95% CI: 0.23, 22.00, *p* < 0.5). There was a significant negative association between living in urban areas and higher oil retinol levels at the 90th percentile (−10.91; 95% CI: −21.27, −0.54, *p* < 0.05), and the effects at other percentiles were smaller and not significant. There was also a small significant negative association between dietary diversity and retinol levels in household cooking oil at the 25th percentile (−0.92; 95% CI: −1.79, −0.04, *p* < 0.05).

### 3.2 Effectiveness of fortification

In the MASAVA project, the SMS fortification intervention may have increased retinol‐binding protein levels in children consuming oil regularly in the intervention districts but had no effect on the prevalence of VAD due to the low coverage of SMS‐produced fortified sunflower oil by the time of the end line survey (Horton et al., 2017; Walters et al., 2018; Walters, 2018). Conversely, the legislation and enforcement of mandatory fortification standards for large‐scale producers had a strong association with the reduction of the prevalence of VAD by 21.4% points in both children and mothers over the 18‐month period in the Shinyanga region (Horton et al., 2017; Walters et al., 2018; Walters, 2018). The plausibility of this effectiveness finding is strengthened by the evidence of Shinyanga's preference for vegetable oil, which is more commonly produced by large‐scale enterprises than sunflower oil in Tanzania, and the significant increase in oil retinol levels after the revised national fortification standards was enacted and enforced. At the current prevalence level, VAD is associated with 770 child deaths and 66,066 DALYs lost each year in Tanzania. If the whole population had access to adequately fortified edible oil and its health effect is extrapolated to the entire country, it is estimated that 305 child deaths and 26,105 DALYs would be averted each year (Table 1).

**Table 1:** Results for the estimated hypothetical mortality and total disability‐adjusted life years (DALYs) averted due to the effect of mandatory fortification across Tanzania

	Conservative		High estimate	
Disease	Mortality attributed to VAD averted	DALYs averted	Mortality attributed to VAD averted	DALYs averted
Mortality				
Measles	11	892	12	1,048
Diarrhoea	294	24,808	349	29,425
Morbidity				
Child		251		293
Maternal		155		130
Total		26,105		30,996

*Note*. VAD: vitamin deficiency. High estimate is based on the use of the alternative effect of fortification equal to a 25.0 and 31.7% point reduction in the prevalence of VAD in children and women from the differences‐in‐differences regression using adequate fortification as the treatment variable.

### 3.3 The costs of fortification

Table 2 contains the unit‐cost data and assumptions that informed this costing analysis. From the societal perspective, the modelled incremental cost of fortification of oil with vitamin A was $0.39, $0.25, and $0.02 per litre sold in 20‐L containers for small‐, medium‐, and large‐scale producers, respectively, compared with the status quo unfortified oil (see Table 3). The purchase and installation of fortification mixing tanks and other equipment are the largest start‐up capital cost for oil producers. When amortized over the volume of fortified oil produced, the equipment capital costs may add up to $0.20 and $0.06 per litre of oil for SMS producers, respectively, but are negligible for large‐scale producers. Large‐scale producers, and potentially medium‐scale producers, have the capacity to collect, clean, and reuse each 20‐L container approximately 10 times to save on packaging costs, whereas small‐scale producers cannot afford the transport costs to reclaim the containers. Consequently, the cost of not being able to recycle—even with an aftermarket sale of the used containers—adds up to an incremental cost of $0.06 per litre for SMS producers.

**Table 2:** Unit cost and quantity data and proxy assumptions and sources used in the costing and cost‐effectiveness analysis of fortification, by scale of producer

	Small‐scale	Low‐cost small‐scale	Medium‐scale	Low‐cost medium‐scale	Large‐scale	
Variable in costing analysis	Amount	Amount	Amount	Amount	Amount	Source
Price per litre of 20‐L container unfortified oil, USD					0.98	(Market Axis Limited, 2016).
Production volume, L, value	5,801	11,155	23,203	44,621	56,121,684	Note: Estimates from SMS monitoring data. The low‐cost assumption is that production can increase to one half current total all oils. Large‐scale estimate from Tanzania Ministry of Trade and Investment, 2016.
Set of three fortification mixing tanks, USD	20,382	2,000	20,382	2,000	22,500	(Horton et al., 2017). Note: From MEDA reporting.
Other installation costs of mixing tanks, USD	8,223	2,831	8,223	2,831	8,223	(Horton et al., 2017). Note: From MEDA reporting.
Number of sets of tanks	1	1	1	1	3	Note: Assumption
Number of years before replacing equipment	25	25	20	15	10	Note: Assumption
Pumping system for premix, USD	250	250	250	250	500	(Fiedler & Afidra, 2010)
Accessories, USD	525	525	525	525	1050	(Fiedler & Afidra, 2010)
Container label redesign cost, USD	262.50	262.50	525.00	525.00	2100	(Fiedler & Afidra, 2010)
Frequency of redesign, years	10	10	5	5	5	Note: Assumption
Training per staff, USD	20	20	20	20	20	(Fiedler & Afidra, 2010)
Number of staff trained	5	5	20	20	150	(Market Axis limited, 2016). Note: 50% average number employees—Large (MEDA)
Frequency of training needed, years	1	1	1	1	1	Note: Assumption
Premix base per litre, USD	0.008127	0.008127	0.008127	0.008127	0.008127	(Harry cummings and associates, 2017). Note: 189,000 TZS per 1,000 L.
Labour for mixing/premix production per litre, USD	0	0	0.0028667	0.0028667	0.0028667	(Harry Cummings and Associates, 2017). Note: 5,000 TZS/person/day × four people for up to 3,000 L/day, none for SMS.
Additional energy used for pumping, stirring, and filling oil in tank per litre, USD	0.00387	0.00387	$0.00387	0.00387	0.00387	(Harry Cummings and Associates, 2017). Note: 90,000 TZS for 1,000 L of oil.
Quality control per litre, USD	0.0000002	0.0000002	0.0000002	0.0000002	0.0000002	(Harry Cummings and Associates, 2017). Note: 123,035 TZS ($55) per 1,000 L.
Net cost of 20‐L container per litre after resale, USD	0.06	0.06	0.06	0.00	0.00	Large‐ and medium‐scale producers can recycle.
Management cost, 10% of all direct costs	10	10	17.5	17.5	17.5	Assumption: Small‐scale has lower fees.
Distributor/retailer fee, 10% of all direct costs	10	10	10	10	10	Assumption: 10% of all direct costs
VAT of 18%	0	0	18	18	18	VAT on large‐ and medium‐scale enterprises
Public sector cost of fortification per MT oil, USD	3.34	3.34	3.34	3.34	1.67	(National Food Fortification Alliance, 2009). Note: Assumption that cost at least two times for SMS.

*Note*. SMSs: small and medium scales; MEDAs: Mennonite Economic Development Associates; VAT; TZS: Tanzania shilling; USD; U.S. dollar; VAT: value‐added tax.

**Table 3:** Incremental cost (IC) and cost‐effectiveness results of vitamin A fortification by producer scale

Type of fortification programme	IC per litre of oil (compared with unfortified oil), $	IC per litre of oil (compared with unfortified oil) as % of price of unfortified oil	Total IC per capita per year, $	Times more expensive IC than LSFF	Cost per DALYs averted (societal), $
Large‐scale fortified	0.02	2.6	0.13		281
Medium‐scale fortified	0.25	28.9	1.36	10.3	2,892
Lower‐cost medium‐scale fortified	0.06	5.82	0.29	2.2	626
Small‐scale fortified	0.39	41.25	2.09	15.8	4,442
Lower‐cost small‐scale fortified	0.13	14.0	0.71	5.4	1,507

*Note*. DALYs: total disability‐adjusted life years; LSFF: Large‐scale food fortification.

The sensitivity analysis on the incremental cost of fortification demonstrated that government policy changes could help make SMS producers more competitive, which may result in improved access to vitamin A for vulnerable populations. Allowing SMS producers to innovate with the use of low‐cost food‐grade plastic or mild steel with epoxy mixing tanks instead of stainless steel tanks as used in the MASAVA project could reduce the incremental cost of fortified oil by as much as $0.08 per litre (Figure 3). Providing medium‐scale producers with a grant to cover the capital cost for fortification equipment would reduce the incremental cost of fortified oil by $0.10 per litre. The introduction of container recycling, which anecdotal evidence from one SMS producer suggested was feasible, may save an additional $0.10 per litre.

**Figure 4:**
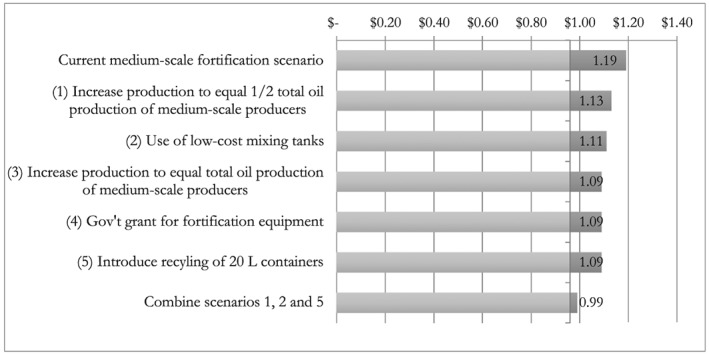
One‐way sensitivity analysis: Estimated cost of fortified sunflower oil per litre by medium‐scale enterprises compared with large‐scale ($) by varying key fortification cost drivers

The incremental cost of fortification for SMS producers is also dependent on the firm's production volume because the cost of capital equipment for fortification can be spread‐out over the entire volume of fortified oil sold during its lifespan. During the MASAVA project, medium‐scale partners fortified approximately one quarter of their total oil production volume. If the production of fortified oil increased to one half of their current total oil production, the incremental cost would decrease by $0.06 per litre. Moreover, increasing the production of fortified oil to equal a firms' current total production of fortified and unfortified oil would decrease the incremental cost further by $0.04 per litre. Increased future production of fortified oil may be plausible because the MASAVA SMS producers scaled‐up fortified oil production rapidly in the last 6 months of the project; SMS producers may be forced to fortify all oil once their exemption from mandatory fortification expires, and consumer demand for domestically produced sunflower oil is projected to increase. With ideal enterprise conditions and certain policy changes, the incremental cost of the fortification may be reduced to as low as $0.06 per litre for medium‐scale producers (Figure 4) and $0.13 for small‐scale producers.

The vast majority of the incremental cost of fortification is incurred by the private sector firms and, subsequently, passed on to consumers through increased purchase price of fortified oils. The increased cost to produce fortified oil, including all amortized capital and recurrent costs, is estimated to be only 2.6, 5.8, and 14.0% higher for large‐scale, medium‐scale, and small‐scale‐produced oil, respectively, compared with unfortified oil. The price of cooking oil can fluctuate by up to 40% depending on the region of Tanzania and increase by up to 30% following harvest season, although SMS producers mostly produce in low‐price season (Market Axis Limited, 2016). The additional annual household expenditure of about $0.67 (1,565 TZS), $1.50 (3,489 TZS), and $3.61 (8,396 TZS) per year for fortified oil produced by large‐, medium‐, and small‐scale enterprises, respectively, compared with unfortified oil seems potentially affordable in this context.

### 3.4 Cost‐effectiveness of fortification

**Figure 5:**
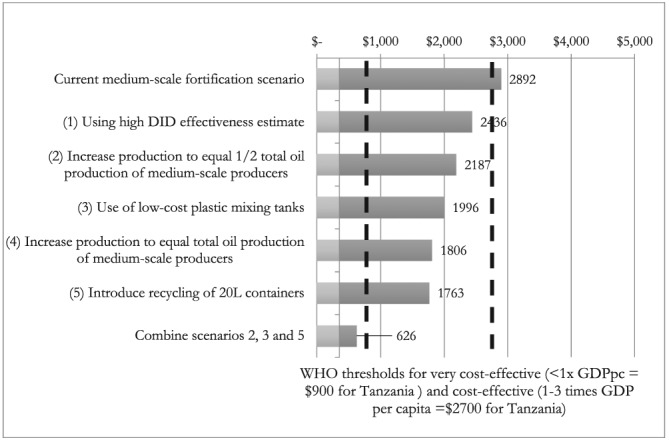
One‐way sensitivity analysis: Estimated cost per total disability‐adjusted life year (DALY) of fortified sunflower oil produced by medium‐scale enterprises compared with large‐scale ($) by varying key fortification factors

From the societal perspective, it is estimated that the cost per DALY averted could be as low as $281 for large‐scale producers compared with unfortified oil (Table 3). If the effect size of fortification of oil with vitamin A by SMS producers could, hypothetically, be assumed to equal the effect from mandatory fortification by large‐scale producers, then, the cost per DALY averted could be as low as $626 and $1,507 for medium‐ and small‐scale producers, respectively. Therefore, fortification would plausibly be considered very cost‐effective using the WHO threshold for reducing VAD (<1 times the GDP per capita) by large‐scale producers and similarly, by medium‐scale producers under certain conditions (Figure 5). The fortification by small‐scale producers may be considered cost‐effective (1–3 times GDP per capita), but this is sensitive to change if enterprise conditions do not improve and favourable policy changes are not enacted.

## 4 DISCUSSION

The rapid increase, from 25 to 58%, in the share of households who consumed edible oil fortified to a level exceeding the mandated minimum is a noteworthy achievement of Tanzania's fortification strategy, programme, and regulatory framework. This study found that legislation of mandatory fortification of oil and its enforcement in line with the new vitamin A fortification quality standards in Tanzania are very cost‐effective in the reduction of VAD and, consequently, could prevent attributable child mortality in the Shinyanga region. With a cost per DALY averted of as low as $281, vitamin A fortification is very cost‐effective relative to the WHO threshold and is comparable with other priority preventative maternal and child health interventions (Horton & Levin, 2016). The estimates of this study, however, show that fortification may not always be as cost‐effective as the previously reported estimates of $18 per DALY averted in Uganda and $35 per DALY averted in the Southern and Eastern Africa region (Fiedler & Afidra, 2010; Horton, 2006). This may be due to context‐specific factors or methodological differences between economic evaluations. Furthermore, the additional 2.2% cost of fortification for large‐scale producers compared with unfortified oil seems manageable, and the increase of annual expenditure of $0.67 per household for fortified oil seems affordable for many Tanzanians.

The MASAVA project intervention demonstrated that it was feasible for SMS producers to produce and distribute fortified sunflower oil with vitamin A. SMS producers may have contributed to modest increases in the retinol‐binding protein levels in children and increased access to fortified oil for certain vulnerable populations. The quantile regression results demonstrated that the region and the household preferences for vegetable oil over sunflower oil in Shinyanga were the primary determinants of access to oil with higher levels of vitamin A. In addition, there was a significant negative association between living in an urban area and oil retinol level at the upper end of the oil retinol distribution. This finding contradicts recent research that suggested that households in rural areas did not have equitable access to fortified oil produced through the large‐scale oil fortification programme in Tanzania (Aaron et al., 2017).

The SMS sunflower oil fortification intervention, however, was unable to reach sufficient coverage to observe a direct effect on the reduction of the prevalence of VAD. Although the pilot project was successful in marketing 140,000 L of fortified sunflower oil, rough estimates suggest that this only covers 1–2% of the demand for sunflower oil in these two regions. Assuming that the health effect of fortified sunflower oil would be equivalent to that of vegetable oil, fortification by medium‐scale producers could be very cost‐effective, and fortification by small‐scale producers could be cost‐effective with favourable policy changes enacted. The low coverage of SMS‐fortified sunflower oil may be due to the trial length and the fact that only three SMS producers participated. Investing in the growth of SMS producers in the sunflower oil sector may have the additional benefits of broadening employment in a greater number of districts, supporting community‐level market systems, and potentially filling gaps in coverage or counter inequities to access of fortified foods created by large‐scale fortification programmes. Policy changes can make SMS fortification become more cost‐effective as the coverage levels increase.

### 4.1 Policy implications

The results of this study suggest that it is in the best interest of the government of Tanzania and its population to continue stewarding investments and the scale‐up of vitamin A fortification across the country. The government can also support SMS producers by removing barriers to entry to the fortification market by providing grants for start‐up capital for fortification equipment (as was done previously for large‐scale producers), permitting the use of less expensive materials for fortification tanks, and permitting use of recycled containers by SMS producers as is already permitted for large‐scale. The government can also invest in a national social marketing strategy to raise awareness of the benefits of fortified oil. Importantly, the government should continue to build capacity in the enforcement of fortification standards to ensure that all producers fortify adequately and protect against risks of toxicity from over‐fortification. The government should also regularly monitor population access to fortified oil and the prevalence of VAD in vulnerable groups in both urban and rural areas. The government of Tanzania's action in 2015, for example, to allow SMS producers to distribute edible oil in 20‐L containers, which enables “by the scoop” sales for poorer households, was essential for the competitiveness of SMS producers.

### 4.2 Limitations

First, some features of the MASAVA project itself, which was in a pilot phase, may have influenced the trial outcomes. For example, the temporary discount on the price of the fortified sunflower oil product for customers and the behaviour change communications strategy implemented to raise awareness for the fortified oil had the potential to increase consumer demand for the fortified products. The influence of both approaches was likely modest because the discount ultimately phased out and radio advertising, which typically has a stronger effect, was not used to avoid compromising the trial control groups (Wu et al., 2019). Ideally, an economic evaluation on fortification would have a longer time horizon and have a mechanism to collect new data on public sector costs of fortification.

Second, seasonality and the annual variation in climatic conditions can affect production output in the agricultural sector, household livelihoods, food security, and availability of foods‐rich in vitamin A. The baseline and end line surveys were undertaken during different seasons; the use of panel data in the effectiveness analysis mitigated against the effects of seasonal and annual changes. Third, inflammation and infection can affect the measurement of retinol binding protein in children and lead to the overestimation of VAD deficiency. The prevalence of malaria infections peaks during rainy seasons and is more likely to affect Shinyanga, which is positioned at a lower altitude and, thus, more favourable for the spread of malaria than the highlands of Manyara. Unfortunately, it was not possible to adjust for the presence of inflammation and infection in the MASAVA project using the new recommended methods for analysis of blood samples because it was not feasible to collect venous blood samples from the sample population (Namaste et al., 2017).

## 5 CONCLUSION

The fortification of edible oils with vitamin A in Tanzania, supported by the government's legislation and enforcement of the mandatory fortification regulation, has been shown to be very cost‐effective in reducing VAD in the Shinyanga region. If the government can institute a few policy changes, sunflower oil fortification by SMS producers can also be cost‐effective and can contribute to the achievement of Tanzania's National 5‐Year Development Plan target to reduce the prevalence of VAD in children under the age of 5–20% by 2025.

## CONFLICTS OF INTEREST

The authors declare no conflicts of interest in relation to this study.

## CONTRIBUTIONS

SH, NS and TM were the principal investigators of the overall project, which made this research possible. SH and TM designed the research instruments. TM and EN led the data collection team. DW analysed the data and wrote the first draft of the paper. All authors reviewed the draft, provided comments, and approved the final draft.
